# An intervention promoting understanding of achievement emotions with middle school students

**DOI:** 10.1007/s10212-020-00498-x

**Published:** 2020-10-02

**Authors:** Daniela Raccanello, Rob Hall

**Affiliations:** 1 Department of Human Sciences, University of Verona, Lungadige Porta Vittoria 17, 37129 Verona, Italy; 2 Sydney, Australia

**Keywords:** Achievement emotions, Understanding emotions, Regulating emotions, Achievement, Middle school students

## Abstract

This study investigated the efficacy of an evidence-based intervention aimed at enhancing middle school students’ understanding of achievement emotions and their regulation. We used the control-value theory of achievement emotions and ability conception from emotional intelligence as a theoretical framework. Participants were 62 students followed longitudinally from seventh to eighth grade, assigned to an experimental or a control condition. Pre- and post-test measures assessed knowledge on achievement emotions, strategies to regulate achievement emotions, native language-related achievement emotions, and grades. The intervention was embedded within the native language domain and included ten units. We focused on a range of ten positive and negative, activating and deactivating emotions, and associated emotion regulation strategies. Compared to the control group, students in the experimental group improved knowledge of emotional lexicon and emotion strategies; they decreased some negative native language-related emotions; and only boys increased native language-related grades. The findings supported the efficacy of the training and were discussed referring to relevant theoretical approaches.

**Electronic supplementary material:**

The online version of this article (10.1007/s10212-020-00498-x) contains supplementary material, which is available to authorized users.

Acquiring an understanding of people’s emotional functioning allows individuals to adapt successfully to social contexts (Denham et al. 2012a; Rivers et al. 2012; Saarni 1999). Within the revised ability model of emotional intelligence (EI; Mayer and Salovey 1997; Salovey and Mayer 1990), this knowledge relates to the third branch of EI, involving reflections on the nature of emotions and the regulation of emotional processes (Goetz and Bieg 2016; Goetz et al. 2005).

An increasing body of research, mainly under the larger umbrella of social and emotional learning (SEL; Brackett and Rivers 2014), has demonstrated that single or multiple components of EI can be fostered through specific interventions. However, such interventions have rarely involved achievement emotions, defined as prospective, concurrent, or retrospective reactions to learning activities or outcomes (Pekrun 2006). In light of the recursive nature of the relations between emotional, motivational, and cognitive dimensions in learning contexts (Linnenbrink-Garcia and Pekrun 2014), enhancing knowledge on achievement emotions and related regulating processes could be an option for facilitating students’ wellbeing and performance (Jones and Bouffard 2012).

We describe a study investigating the efficacy of a pilot evidence-based intervention aimed at enhancing middle school students’ understanding of achievement emotions and their regulation. We used the control-value theory (CVT; Pekrun 2006; Pekrun and Perry 2014) as the main theoretical background.

## Emotions in learning contexts

The CVT has given particular impetus to research into the role of emotions aroused in the context of formal learning, with attention to both activities and outcomes (Pekrun 2006; Pekrun and Perry 2014). Achievement emotions have at least two underlying dimensions, i.e., valence (positive and negative) and activation (activating and deactivating emotions; Feldman Barrett and Russell 1998). Therefore, emotions can be categorized as positive activating (e.g., enjoyment), positive deactivating (e.g., relaxation), negative activating (e.g., anxiety), and negative deactivating (e.g., boredom). An increasing amount of data has documented the relations between these four types of emotions and achievement, through the mediation of cognitive and motivational factors. Research reveals positive and negative associations for positive activating emotions and negative deactivating emotions, respectively, with contradictory findings for positive deactivating emotions and negative activating emotions. In addition, the model assumes that cognitive beliefs about control and value play a role as antecedents of emotion, with factors such as performance being outcomes of emotions. It also assumes that both emotions and their antecedents are organized in domain-specific ways, namely that they differ according to subject domains or sub-domains such as mathematics, science, or native language.

The CVT offers stakeholders such as teachers, parents, or psychologists the potential to foster success at school by intervening in ways that are assumed to influence later performance. However, to date, scarce attention has been paid to the possibility of enhancing school performance by focusing on students’ understanding of emotions. As an exception, Goetz and colleagues (Goetz and Bieg 2016; Goetz et al. 2005) integrated some components of EI within the CVT, and proposed the PEILAS model (for the Promotion of EI in Learning and Achievement Situations), according to which achievement emotions are linked to three of the four components of EI. Specifically, instructional content about emotions and strategies for regulating them would be key resources to improve learners’ EI skills. Against this background, it is possible to promote those cognitive abilities that enable a student to behave in an emotionally intelligent way within a learning context. One example might be involving the students in activities aimed at widening their emotion vocabulary, e.g., games through which they can increase the number of adequate terms that they are able to use to describe emotions. Another example could be involving them in group projects or other activities that help them become acquainted with emotion regulation (ER), that is “the ability to decrease, maintain or increase one’s emotional arousal to facilitate engagement with the context” (Molina et al. 2014, p. 625). They could work collaboratively to identify emotions and effective emotion regulation strategies relevant to their learning context, such as test-related emotions.

## Understanding of emotions

During development, individuals strengthen their abilities to process and understand the role of emotions in a variety of everyday contexts (Rivers et al. 2012). The understanding of emotions, as a complex ability comprising sub-components, gradually develops from preschool to school years (Pons et al. 2010). Understanding of emotions develops across three hierarchical periods (Pons et al. 2004): at about five, children understand public elements of emotions, relating to expression and external causes; at about seven, they understand the mentalistic nature of emotions in terms of differences between expressed and underlying emotions; at about nine, they understand their reflexive connotation, including knowledge on methods that can be used to regulate emotions (Denham et al. 2012a, 2012b; Harris 2008; Pons et al. 2004; Saarni 1999). In parallel with the increasing awareness of internal states we see a developing ability to understand and use a psychological lexicon to verbally label emotional states. This leads to both improved communicative processes and higher elaboration of emotional experiences (Harris 2008).

The understanding of emotions is linked to the use of ER in everyday life (Cicchetti et al. 1995). As with more general regulation processes, ER implies the reduction of the difference between an actual and a desired state (Goetz and Bieg 2016). Within learning, emotions can be perceived as disruptive, resource-consuming, or as interfering with the process of goal attainment (Jacobs and Gross 2014).

Developmental trends in the ability to regulate emotions have been described. During the school years, support-seeking strategies become more self-reliant rather than adult-reliant, problem-solving strategies become more based on planned rather than instrumental actions (i.e., actions performed for their consequences or outcomes), and distraction is characterized also by cognitive strategies rather than solely behavioral strategies (Zimmer-Gembeck and Skinner 2011). Elementary school students are already able to use cognitive reappraisal strategies, as “a form of cognitive change that involves construing a potentially emotion-eliciting situation in a way that changes its emotional impact”; this is associated with adaptive development at the affective, cognitive, and social level (Gross and John 2003, p. 349; Jacobs and Gross 2014). Changes in ER from elementary to middle school have also been documented in connection with changes in some achievement emotions, with associations between enjoyment and problem-solving, coping and palliative ER, and between boredom and avoidant coping and anger-related ER (Vierhaus et al. 2016). The recent integrated model of ER in achievement situations has discussed the complexity of the relation between ER strategies and achievement emotions, depending on aspects such as object foci, time frames, and appraisal patterns (Harley et al. 2019). Indeed, these authors stressed the key role of adults’ instructional interventions about using reappraisal strategies to pre-adolescents, who still do not have the cognitive abilities to master such cognitively demanding strategies autonomously.

Notwithstanding the relevance of the understanding of emotions for school performance (Denham et al. 2012a), the links between the two constructs still remain underexplored. Some empirical evidence indicated that understanding of emotions promotes current and later school success. However, most of the studies focused on preschool children (Denham et al. 2012a, 2012b; Izard et al. 2001; Rhoades et al. 2011) and less is known about elementary to university students, for whom inconsistent findings have emerged (Collins and Novicki 2001; Di Fabio and Palazzeschi 2009; Garner 2010; Márquez et al. 2006; Mavroveli and Sanchez-Ruiz 2011; O’Connor Jr. and Little 2003; Rivers et al. 2012). Nevertheless, on the whole, the relations between ability-based EI, and understanding emotions, managing abilities, and academic achievement have been confirmed; however, findings are mixed, especially after controlling for intelligence and personality (Allen et al. 2014).

## Intervention programs on emotions in learning contexts

At the beginning of middle school, pre-adolescents already manage the psychological lexicon appropriate to describe how they and other persons feel, and they are also increasingly acquainted with a range of strategies for regulating emotions (Beyers and Çok 2008; Labouvie-Vief et al. 1995). Some research highlighted the decreasing intensity of positive emotions and increasing intensity of negative emotions that can occur during schooling (Raccanello et al. 2013). Planned interventions that focus on assisting children to understand and manage their emotions associated with their levels of achievement might mitigate future problems that result, for example, in dropping out of school (Eccles and Roeser 2009). However, to date, little attention has been paid to the development of interventions of this kind, notwithstanding the wide diffusion of SEL programs (Brackett and Rivers 2014).

SEL programs aim at fostering the integration of cognitive, emotional, and behavioral dimensions to increase awareness, responsible decisions, and management of behaviors (Brackett and Rivers 2014). A meta-analysis of social and emotional development including more than 200 studies with elementary to high school students, documented their positive impact not only on social and emotional skills, but also on improved school success, social relations, and diminished problem behaviors (Durlak et al. 2011). However, effect sizes were generally weak, probably because SEL programs do not focus specifically on academic competencies. The meta-analysis did identify some factors that moderate the efficacy of the programs, such as the intervention format or the reliance on specific characteristics in the training procedure. Interventions conducted by the school staff were successful across the whole range of measured outcomes, while they were only partially successful when delivered by non-school personnel such as university researchers or outside consultants. This might be predicted on the basis of the assumptions underlying sociocultural approaches (Boekaerts and Corno 2005). In contrast, the use of Sequenced, Active, Focused, and Explicit (SAFE) training procedures resulted in statistically significant progress for all the indicators. Sequenced procedures imply a range of linked activities coordinated to achieve the objectives of the programs; active techniques refer to students’ proactivity emerging in cooperative learning settings; focused and explicit programs include at least some activities on personal and specific rather than general social and emotional skills.

Notwithstanding the diffusion of EI programs within schools, two main shortcomings of many interventions could be identified, namely the lack of a clear definition of EI and the focus on social emotions rather than achievement emotions (Goetz and Bieg 2016; Goetz et al. 2005). However, we know that middle school students’ achievement emotions can be influenced in ways that produce positive direct and indirect effects on performance (Buff et al. 2011). We also know that understanding of emotions can be enhanced with specific training (Sprung et al. 2015), for example through the promotion of a wider emotional lexicon amongst pre-schoolers (Grazzani Gavazzi and Ornaghi 2011). Further, we know that intervening on appraisals of emotions influences achievement emotions, for example with attributional retraining strategies resulting in benefits for adaptive attribution-related emotions for university students (Hamm et al. 2014). Nevertheless, to our knowledge interventions addressing middle school students’ understanding of achievement emotions have never been proposed and tested.

## Aims and hypotheses

We investigated the efficacy of a pilot intervention aimed at promoting middle school students’ understanding of achievement emotions, embedded within the domain of native language. This choice reflected the assumptions about the domain-specificity spelt out in the CVT as well as experiences from interventions using domain-specific measures within self-regulation programs (Boekaerts and Corno 2005).

Students belonging to an experimental group experienced the intervention, while at four time-points we assessed outcomes from both the experimental and a control group. The intervention was designed to facilitate the use of a psychological lexicon appropriate for describing achievement emotions (first phase) and of strategies for regulating negative achievement emotions (second phase). The outcome measures were assessed just before (T1) and after (T2) the first phase, and just before (T3) and after (T4) the second phase.

### Hypothesis 1

We expected that students in the experimental group would show improvements in their ability to access and report (H1a) the psychological lexicon of achievement emotions at three time-points (T2, T3, T4), and (H1b) functional ER and reappraisal strategies in the short term (T4).

### Hypothesis 2

We explored whether the effects of the intervention extended to the intensity of the achievement emotions associated with native language, at three time-points (T2, T3, T4). We expected higher intensity of positive emotions and lower intensity of negative emotions for the experimental compared to the control group.

### Hypothesis 3

We explored whether the effects of the intervention were associated with improvements in native language-related performance, at two time-points (T2, T4). We expected higher grades for the experimental compared to the control group.

## Methods

### Participants

The sample comprised 62 students from a middle school in Northern Italy, followed longitudinally from the seventh to the eighth grade. It was divided into an experimental group that participated in the intervention (*n* = 33; *M* = 12.7, range 12.2–13.1, at the beginning of the study; 46% females) and a control group that did not participate (*n* = 29; *M* = 12.7, range 12.2–13.1; 45% females). Each group was split across two classes to fit with the logistics of the native language (Italian) curriculum at the school. The students were allocated to the intervention or the control group depending on school constraints. Specifically, when we began the study, there were three teachers who were involved in the native language lessons for seventh-graders in the school whose head had given her authorization for this research. Among them, only one teacher could conduct the intervention during her lessons, and she was teaching in two classes, i.e., the two classes included in the experimental group. An additional 20 students participated, but they were excluded from the final sample for reasons of physical or cognitive disabilities (such as physical disorders, emotional and behavioral disorders, etc.) or because they did not attend at least one of the four time-points of the intervention. The study was carried out following APA ethical guidelines and was approved by the Local Ethical Committee of the Department of Human Sciences, University of Verona. All the students participated after their parents had signed a consent form.

The groups were similar for family characteristics such as fathers’ age (experimental group: *M* = 46 years, range 36–54; control group: *M* = 50 years, range 39–63), mothers’ age (experimental group: *M* = 43 years, range 33–51; control group: *M* = 44 years, range 34–54), and family composition (91% and 96% of children, respectively, for the experimental and the control group, had two-parent families), and they came from a wide range of socio-economic background.

### Intervention

The intervention comprised an eight-unit first phase (March–May, 2015) when the students were seventh-graders and a two-unit second phase (December, 2015) when they were eighth-graders. The units focused on understanding of achievement emotions and strategies to regulate them. We defined the key constructs of achievement emotions and ER strategies and aimed at extending the emotion vocabulary to describe them (Goetz and Bieg 2016; Goetz et al. 2005), through activities based on the knowledge and experience of a researcher in educational psychology whose focus is emotions, and a native language teacher. The units were embedded within the native language domain. Activities focused on verbal recognition or verbal production abilities as sub-components of understanding emotions, considering recognition abilities as prerequisites of the production abilities. All activities were developed ad hoc, based on the psychological literature. See Supplementary materials for a detailed description of activities, objectives, procedure, and materials.

The first eight units contained one activity, while units nine to ten consisted of three activities. Activities were presented weekly, over a 1-h session for units one to eight and a 1.5-h session for units nine to ten. The students worked individually or in dyads and small groups to foster a cooperative learning setting. Whenever a student missed one unit, s/he was debriefed about its contents before the following one. Thirteen students missed one unit; two students missed two units; one student missed three units; and one student missed four units. First-phase activities were guided by the native language teacher, while second-phase activities were guided by a researcher in educational psychology (Goetz and Bieg 2016).

#### First phase

Activities aimed at developing students’ abilities to recognize and use the psychological lexicon related to ten achievement emotions, i.e., three positive activating emotions (enjoyment, hope, pride), two positive deactivating emotions (relief, relaxation), three negative activating emotions (anxiety, anger, shame), and two negative deactivating emotions (boredom, hopelessness; Kleine et al. 2005). At the beginning of the intervention, the teacher explained what achievement emotions are, defining them according to the CVT. The activities of the first eight units were linked to four textual genres, chosen in accordance with the guidelines on Italian national curricula: narrative (activities 1, 2), expressive (3, 4), dialogue (5, 6), and social network language (7, 8). For each genre, the first unit always focused on verbal recognition abilities, while the second on verbal production abilities. For example, when training the students to recognize the emotional lexicon in narrative texts (unit 1), we presented ten extracts from ten narrative books and we asked them to name the main emotion described, to identify the emotional terms, to state whether the emotion was an achievement emotion or not, and to report its causes. When training them in using the emotional lexicon in narrative texts (unit 2), we asked them to produce two brief narrative texts on a positive and a negative achievement emotion, respectively. For the expressive genre, we asked them to identify the three synonyms describing the achievement emotion expressed in diary texts which had been developed ad hoc, within a list of 30 terms (Raccanello et al. 2013; unit 3), and then we prompted them to reflect on their experience on achievement emotions through open-ended questions (unit 4). For dialogue, we asked them to label the main emotion within each of ten comic strips and to identify its cause (unit 5), and then to perform a brief sketch representing two events focused on two achievement emotions randomly assigned (unit 6). For social network language, we asked them to develop a shared code to represent the ten target achievement emotions using WhatsApp emoticons (unit 7), and then to write dialogues including at least two achievement emotions with corresponding labels and emoticons, using the shared code (unit 8).

We selected and/or wrote all the materials (e.g., written texts, emoticons, etc.) focusing on one of the ten achievement emotions. Before using them, they were independently coded for their adequacy by a researcher expert in emotions. Whenever the intended emotion was not properly identified, alternative materials were proposed, coded again for their adequacy.

#### Second phase

Activities aimed at developing students’ abilities to access and report verbally ER strategies related to achievement emotions. Focusing on ER was particularly appropriate for eighth-graders, who had to cope with their first state exam at the end of the school year, marking the change from Italian middle to high school instruction. Definitions of ER strategies were in line with the literature, highlighting that in some settings ER strategies can be functional and effective, together with recognizing that their adaptivity varies according to both individual and contextual factors (Molina et al.^ 2014^; Phillips and Power 2007). Again, the units training recognition abilities and production abilities were alternated. The activities aimed at: identifying emotions in need of ER in different school settings, i.e., during lessons, before/during/after tests, and during homework, writing a list of emotional terms (activity 9); recognizing functional ER strategies among a list of 19 ER strategies (Phillips and Power 2007), sharing students’ choices through the construction of a class poster (activity 10); reporting verbally (activity 11) and negotiating (activity 12) functional ER strategies in different school settings, again constructing posters in small groups, to be shared with the whole class; becoming familiar with an instrument facilitating access to functional strategies in everyday life, i.e., a laminated bookmark including the picture of a traffic light corresponding to three steps for ER, namely *stop*, *think*, and *start again* (activity 13); and using the strategies in a simulated situation performing brief sketches (activity 14). Particular attention was paid to fostering reappraisal strategies (Gross and John 2003). For ethical reasons, after T4 the control group participated to units nine to ten according to a wait-list design.

### Outcome measures

We gathered pre- and post-intervention measures at four time-points, in which we assessed students’ ability to use the psychological lexicon of achievement emotions and native language-related achievement emotions. We also measured students’ ability to verbally report ER strategies at T3 and T4, and we gathered data on their performance at T1, T2, and T4.

#### Emotion understanding

The efficacy of the intervention was evaluated with two ad hoc tests measuring students’ ability to produce achievement emotion terms for the first phase (i.e., achievement emotions vocabulary task) and to report ER strategies for the second phase (i.e., ER list task). In the achievement emotions vocabulary task, we asked the students to write as many terms referring to emotions that can be felt at school as they could in a 5-min period. In the ER list task, we asked to write as many ER strategies for diminishing the intensity/duration of five negative achievement emotions which can be felt in different settings (i.e., anxiety before a test, anger and sadness after a bad grade, shame during a test, boredom during a lesson) as they could in a 15-min period.

We coded the number of (a) achievement emotions (Kleine et al. 2005; Pekrun 2006; Pekrun and Perry 2014), (b) functional ER strategies (Phillips and Power 2007), and (c) reappraisal strategies (Gross and John 2003). A first judge coded 100% of the protocols, while a second judge coded 30% of them (mean agreement for achievement emotions vocabulary task/ER list task: 97%/91%). Disagreements were resolved through discussion.

#### Native language-related achievement emotions and performance

The students completed the Achievement Emotions Adjective List questionnaire (AEAL; Raccanello 2015; Raccanello et al. 2015a, b, 2020a, b), developed on the basis of the CVT. We presented 30 words (mainly adjectives) describing how they might feel during native language lessons, to be evaluated on a 7-point Likert scale (1 = *not at all* and 7 = *completely*). The 30 words related to ten achievement emotions, including three positive activating emotions (enjoyment, hope, pride), two positive deactivating emotions (relief, relaxation), three negative activating emotions (anxiety, anger, shame), and two negative deactivating emotions (boredom, hopelessness). Alpha scores for each emotion ranged from 0.70 to 0.93 (except hope at T1: *α* = 0.57; Table 1).

**Table 1:** Alpha scores (α), means (M), and standard deviations (SD) for the intensity of native language-related achievement emotions, separately by group (experimental, control) and time (1, 2, 3, 4), and intercorrelations with performance

Variable	Time	*α*	Experimental group: *M(SD)*	Control group: *M(SD)*	1	2	3	4	5	6	7	8	9	10
1. Enjoyment	1	0.84	2.95(1.12)	3.06(0.99)										
	2	0.85	3.04(0.87)	2.90(1.06)										
	3	0.83	3.15(0.91)	3.21(0.81)										
	4	0.81	2.97(0.91)	2.94(0.86)										
2. Hope	1	0.57	3.11(1.13)	3.33(0.94)	.56***									
	2	0.78	2.8 (0.94)	3.26(0.87)	.28*									
	3	0.82	3.13(0.96)	3.18(0.82)	.56***									
	4	0.85	2.73(0.95)	3.10(0.94)	.51***									
3. Pride	1	0.82	2.95(0.87)	3.36(0.96)	.74***	.70***								
	2	0.82	2.90(0.81)	3.01(0.96)	.72***	.56***								
	3	0.90	2.89(0.90)	3.14(0.96)	.61***	.73***								
	4	0.85	2.65(0.95)	3.02(0.77)	.65***	.77***								
4. Relief	1	0.85	3.09(1.05)	3.02(0.71)	.72***	.55***	.68***							
	2	0.84	2.87(0.87)	2.83(0.93)	.65***	.35***	.76***							
	3	0.85	3.02(1.08)	3.01(0.76)	.51***	.62***	.59***							
	4	0.86	2.78(0.97)	2.83(0.82)	.54***	.75***	.62***							
5. Relaxation	1	0.75	3.42(1.00)	3.32(0.95)	.51***	.49***	.57***	.68***						
	2	0.74	3.41(0.72)	3.16(0.93)	.57***	.15	.51***	.61***						
	3	0.83	3.70(0.88)	3.35(0.83)	.48***	.41**	.43**	.64***						
	4	0.75	3.56(0.92)	3.25(0.61)	.43***	.32*	.28*	.52***						
6. Anxiety	1	0.70	1.98(0.89)	1.82(0.86)	− .38**	− .38**	− .41**	− .26*	− .42**					
	2	0.72	1.66(0.71)	2.07(0.82)	− .22	.19	− .05	− .21	− .31*					
	3	0.77	1.90(0.88)	1.95(0.79)	− .32*	− .06	− .09	− .32*	− .42*					
	4	0.85	1.63(0.74)	2.09(0.88)	− .12	− .01	− .02	− .24	− .49***					
7. Anger	1	0.91	1.56(0.79)	1.70(1.02)	− .34**	− .42**	− .32*	− .28*	− .31*	.42**				
	2	0.91	1.46(0.85)	2.16(1.20)	− .46***	.01	− .39**	− .43***	− .54***	.70***				
	3	0.82	1.59(0.70)	1.82(0.84)	− .31*	− .33**	− .32*	− .42**	− .62***	.64***				
	4	0.83	1.48(0.71)	1.89(0.80)	− .34**	− .17	− .22	− .30*	− .50***	.71***				
8. Shame	1	0.85	1.42(0.63)	1.63(0.89)	− .35**	− .15	− .35**	− .22	− .36**	.67***	.23			
	2	0.73	1.36(0.56)	1.55(0.58)	− .08	.05	− .06	− .17	− .16	.58***	.51***			
	3	0.87	1.40(0.68)	1.55(0.62)	− .26*	− .04	− .10	− .26*	− .42**	.64***	.49***			
	4	0.87	1.39(0.69)	1.64(0.78)	− .05	− .08	− .03	− .02	− .40**	.81***	.57***			
9. Boredom	1	0.92	2.74(1.26)	2.53(1.24)	− .50***	− .45***	− .38**	− .37**	− .45***	.62***	.64***	.41***		
	2	0.93	2.25(1.13)	3.13(1.40)	− .64***	.04	− .48***	− .43**	− .47***	.65***	.76***	.42**		
	3	0.93	2.47(1.15)	2.61(1.18)	− .61***	− .43**	− .56***	− .51***	− .46***	.55***	.67***	.40**		
	4	0.92	2.60(1.31)	2.83(1.16)	− .43***	− .34**	− .39**	− .38**	− .31*	.53***	.65***	.48***		
10. Hopelessness	1	0.86	1.71(0.90)	1.54(0.83)	− .44***	− .59***	− .44***	− .43***	− .56***	.69***	.72***	.36**	.72***	
	2	0.85	1.35(0.62)	2.01(1.07)	− .44***	.11	− .32*	− .38**	− .42**	.65***	.71***	.54***	.71***	
	3	0.81	1.60(0.76)	1.83(0.82)	− .43***	− .29*	− .36**	− .47***	− .50***	.62***	.67***	.64***	.68***	
	4	0.87	1.57(0.83)	1.84(0.84)	− .30*	− .21	− .24	− .38**	− .40**	.73***	.77***	.66***	.70***	
11. Performance	1	-	7.06(1.09)	7.21(1.21)	.05	.25	.12	.01	.22	− .44***	− .27*	− .23	− .36**	− .37**
	2	-	7.48(1.09)	7.45(1.15)	.38**	.23	.33**	.22	.25*	− .20	− .30*	− .30*	− .28*	− .41**
	4	-	7.12(0.99)	7.10(1.29)	.23	− .04	.04	− .09	.17	− .26*	− .14	− .12	− .20	− .13

*N* = 62. For columns 1 to 10, correlations refer to the same time as indicated in the row

*p < .05; **p < .01; ***p < .001

Administrative staff provided the records of students’ grades in native language, as included in their report card, assigned at the end of the first (T1) and second term (T2) of the seventh grade and at the end of the first term of the eighth grade (T4). In the Italian education system, grades range from 1 (very poor) to 10 (excellent), with grades from 1 to 5 considered as fail, 6–7 as pass, 8 as good, and 9–10 as excellent. Therefore, higher grades equal to higher achievement.

### Data analyses

We used SPSS version 21.0 for Windows to run all the analyses, namely Pearson bivariate correlations, *t* tests, and analyses of variance (ANOVA). For post hoc pairwise comparisons, Bonferroni correction was used. The level of significance was *p* < .05. In preliminary analyses, we explored the role of gender, and we included it as a between-subjects factor whenever it resulted in statistically significant interactions. For measures on emotion understanding, there were no missing data. For the other measures, we conducted the Little MCAR test to check the distribution of the missing data, which returned a non-significant result, *Χ*^*2*^(6159) = 0.001, *p* = 1.000. Given that the missing data were distributed at random, and that the percentage of occurrences was very low (i.e., < 4% for 5 items, < 2% for 33 items, and 0% for the others), we conducted the analyses with listwise exclusion. All significant effects and interactions are reported in Table 2. Sample size, although relatively small due to the nature of the research as a pilot study and the length of the intervention, was however in line with the literature (Schonert-Reichl et al. 2015; Wilson VanVoorhis and Morgan 2007).

**Table 2:** *F* values (*F*), degrees of freedom (*df*), *p* values (*p*), and eta square (*η*^*2*^) for significant effects and interactions, for each ANOVA

Dependent variable	Significant factors or interactions	*F*	*df*	*p*	*η*^*2*^
Number of emotions	Group Time Group × time	10.69 34.00 5.23	1,60 3,180 3,180	.002 < .001 .002	0.15 0.36 0.08
Number of ER strategies	Group Group × time	4.666 20.224	1,60 1,60	.035 < .001	0.07 0.25
Number of reappraisal strategies	Time Group × time	4.05 9.03	1,60 1,60	.049 .004	0.06 0.13
Intensity of native language-related achievement emotions	Group Emotion Group × time Group × time × emotion	4.39 60.74 3.23 1.99	1,60 9,540 3,180 27,1620	.040 < .001 .024 .002	0.07 0.50 0.05 0.03
Grades in native language	Gender Time Gender × time Group × gender × time	7.99 6.44 3.97 5.84	1,58 2,116 2,116 2,116	.006 .002 .022 .004	0.12 0.10 0.06 0.09

## Results

### Emotion understanding

#### Achievement emotions vocabulary task

**Fig. 1:**
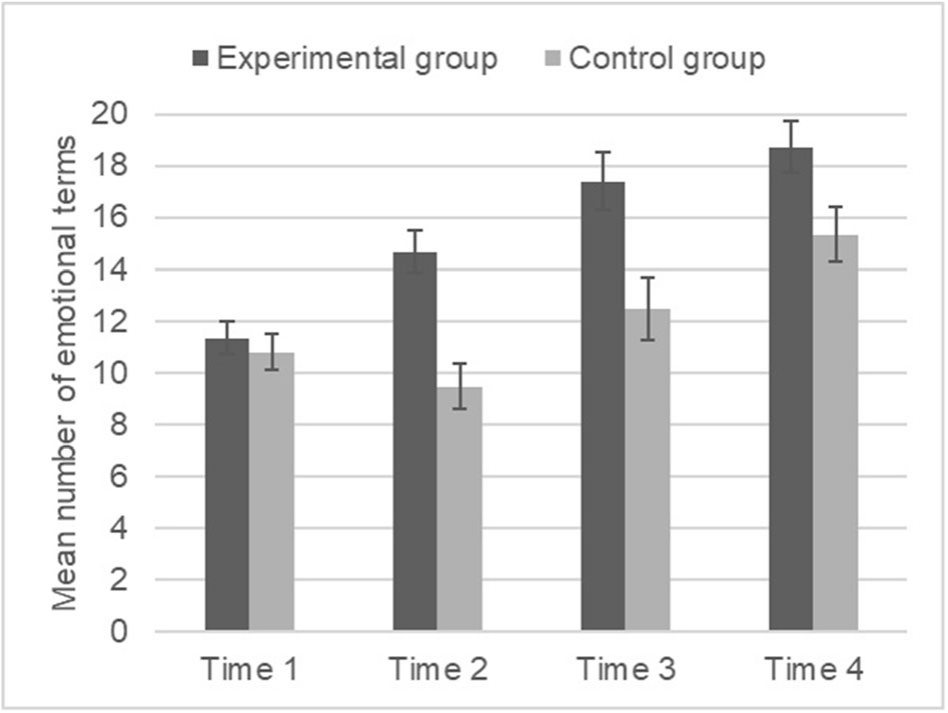
Means (M) and standard errors (SE) of achievement emotions, at each time-point, separately by group (experimental, control)

We carried out a 2 × 4 (group [experimental, control] × time [1, 2, 3, 4]) repeated-measure ANOVA on the number of emotions. It revealed a statistically significant effect of group, with higher scores for the experimental (*M* = 15.53, *SD* = 4.52) compared to the control group (*M* = 12.02, *SD* = 3.85). Also, a statistically significant effect of time emerged: Pair comparisons (all *p*s < .001) revealed that emotions were less frequent for seventh (T1: *M* = 11.08, *SD* = 3.71; T2: *M* = 12.23, *SD* = 5.34) compared to eighth-graders (T3: *M* = 15.10, *SD* = 6.88; T4: *M* = 17.15, *SD* = 6.02). These effects were moderated by a group × time interaction. Independent sample *t* tests, separated by time, indicated that the groups did not differ at T1, but they differed at T2, *t*(60) = 4.36, *p* < .001, *d* = 1.13 (experimental: *M* = 14.67, *SD* = 5.46; control: *M* = 9.45, *SD* = 3.63), T3, *t*(60) = 2.98, *p* = .004, *d* = 0.77 (*M* = 17.39, *SD* = 7.68; *M* = 12.48, *SD* = 4.72, respectively), and T4, *t*(60) = 2.28, *p* = .026, *d* = 0.58 (*M* = 18.73, *SD* = 5.30; *M* = 15.35, *SD* = 6.37), with higher scores for the experimental group. This suggested that the intervention was successful in terms of increasing the ability to access and report psychological lexicon referred to achievement emotions, at both short time and long time (Fig. 1).

#### Emotion regulation list task

We performed a 2 × 2 (group [experimental, control] × time [3, 4]) repeated-measure ANOVA on the number of ER strategies. Group was significant, with a higher number of strategies for the experimental (*M* = 10.12, *SD* = 4.93) compared to the control group (*M* = 8.33, *SD* = 4.27). Also group × time was significant. Independent sample *t* tests, separated by time, with group as the independent variable, revealed differences only at T4, with ER strategies, *t*(60) = 2.78, *p* = .007, *d* = 0.89 (experimental: *M* = 11.55, *SD* = 5.88; control: *M* = 7.83, *SD* = 4.45), higher for the experimental group. These findings revealed that the intervention contributed to increase the ability to have access and verbally report strategies to regulate achievement emotions.

We also checked for differences in reappraisal, conducting a 2 × 2 (group [experimental, control] × time [3, 4]) repeated-measure ANOVA on the number of reappraisal strategies. We found a statistically significant effect of time, with scores increasing from T3 (*M* = 4.44, *SD* = 2.96) to T4 (*M* = 5.76, *SD* = 4.79), in turn moderated by a group × time interaction. Paired *t* tests, separated by group, indicated that reappraisal increased with time only for the experimental group, *t*(32) = − 3.11, *p* = .004, *d* = − 0.54 (T3: *M* = 3.67, *SD* = 2.97; T4: *M* = 5.76, *SD* = 3.30). This result showed that the intervention fostered abilities to list reappraisal strategies for regulating negative emotions.

### Native language-related achievement emotions and performance

#### Correlations

We conducted Pearson correlations among native language-related achievement emotions and performance at each time-point (Table 1). We observed significant positive correlations between positive emotions (except hope and relaxation at T2), as well as between negative emotions (except anger and shame at T2). All the significant correlations between the positive and negative emotions were negative in sign. Significant correlations between emotions measured at the same time-point, occurred consistently between enjoyment, relief, relaxation, anger, boredom, and hopelessness (the percentages of statistically significant correlations to the total number of correlations between positive and negative emotions were 80% for enjoyment; 75% for relief; 95% for relaxation; 85% for anger; 95% for boredom; and 85% for hopelessness), while correlations were negative only for about half of the cases when correlations included hope (40%), pride (60%), anxiety (50%), and shame (35%).

Among positive emotions, performance positively correlated with enjoyment, pride, and relaxation at T2. Regarding negative emotions, performance negatively correlated with anxiety, anger, boredom, and hopelessness at T1; with anger, shame, boredom, and hopelessness at T2; and with anxiety at T4.

#### Native language-related achievement emotions

We performed a 2 × 4 × 10 (group [experimental, control] × time [1, 2, 3, 4] × emotion [enjoyment, hope, pride, relief, relaxation, anxiety, anger, shame, boredom, hopelessness]) repeated-measure ANOVA on the intensity of native language-related achievement emotions. There was a statistically significant effect of group, with lower scores for the experimental (*M* = 2.41, *SD* = 0.30) compared to the control group (*M* = 2.56, *SD* = 0.28). Also, the main effect of emotion, the group × time interaction, and the group × time × emotion interaction emerged as significant. We conducted independent sample *t* tests, separated by time, with group as the independent variable, on the intensity of each achievement emotion. We found statistically significant differences at T2 for anxiety, *t*(60) = − 2.13, *p* = .038, *d* = − 0.54, anger, *t*(60) = − 2.65, *p* = .010, *d* = − 0.67, boredom, *t*(60) = − 2.72, *p* = .009, *d* = − 0.69, and hopelessness, *t*(60) = − 3.00, *p* = .004, *d* = − 0.76, and at T4 for anxiety, *t*(60) = − 2.26, *p* = .028, *d* = − 0.57, and anger, *t*(60) = −2.09, *p* = .041, *d* = − 0.54. In all these cases, the intensity was lower for the experimental compared to the control group (Table 1).

#### Native language-related performance

We carried out a 2 × 2 × 3 (group [experimental, control] × gender [male, female] × time [1, 2, 4]) repeated-measure ANOVA with grades in native language as dependent variables. The ANOVA revealed statistically significant effects of gender and time. Females (*M* = 7.60, *SD* = 0.96) had higher grades than males (*M* = 6.94, *SD* = 0.90), while grades were higher at T2 (*M* = 7.47, *SD* = 1.11) compared to T1 (*M* = 7.13, *SD* = 1.14) and T4 (*M* = 7.11, *SD* = 1.13) (pairwise comparisons, *p* < .01). Also, a statistically significant gender × time interaction and a statistically significant group × gender × time interaction were found. To explore the three-way interaction further, we carried out independent sample *t*-tests separated by gender, with grade at the three time-points as the dependent variable. At T4, only males belonging to the experimental group (*M* = 7.33, *SD* = 0.84) had higher grades, *t*(32) = 2.14, *p* = .040, *d* = 0.72, compared to the control group (*M* = 6.63, *SD* = 1.09). Grades did not differ according to group nor at T1 neither at T2.

## Discussion

Together with expressing adaptive emotions and regulating them, knowledge and understanding of emotions helps to “grease the cogs” of successful school experiences, aside from being associated with healthier psychological functioning and greater social competence (Denham et al. 2012a; Rivers et al. 2012). Children acquire this ability early and gradually refine it as a key resource in the complex process of continuously adapting to changing environments (Harris 2008; Saarni 1999). Focusing on Pekrun’s CVT of achievement emotions (Pekrun 2006; Pekrun and Perry 2014), Goetz and colleagues highlighted the potential to foster knowledge on emotions in learning contexts as an alternative way to influence achievement emotions and proposed the PEILAS model (Goetz and Bieg 2016; Goetz et al. 2005). However, notwithstanding the current diffusion of SEL programs at any school level (Brackett and Rivers 2014), little attention has been paid to testing the efficacy of evidence-based programs believed to foster the understanding of achievement emotions using rigorously designed experimental studies and age-specific materials and assessment instruments.

Conceptualizing this ability within the revised ability model of EI (Mayer and Salovey 1997; Salovey and Mayer 1990), we explored whether a pilot intervention promoting middle school students’ knowledge about achievement emotions and strategies to regulate them was efficacious and generalized its effects to academic performance. The intervention, assigning students to an experimental or a control group, was embedded within the native language domain in light of the domain-specific assumptions of the CVT. Students of the same age as those engaged in our study have the potential to benefit from such interventions in light of their increased cognitive, meta-cognitive, linguistic, and emotional abilities (Beyers and Çok 2008; Giedd 2008; Gross and John 2003; Jacobs and Gross 2014; Labouvie-Vief et al. 1995; Vierhaus et al. 2016; Zimmer-Gembeck and Skinner 2011).

Our preliminary findings demonstrated that a relatively short intervention on knowledge of achievement emotions and strategies for regulating them, embedded within the domain of Italian lessons, can be implemented successfully, yielding positive outcomes for middle school students’ wellbeing and performance. In the experimental compared to the control group, there were improvements in both the understanding of emotions and native language-related achievement emotions and performance. In devising the intervention activities, we partially followed Goetz and colleagues’ suggestions about ways to promote the understanding of emotions as the reflection component of EI (Mayer and Salovey 1997; Rivers et al. 2012; Salovey and Mayer 1990). As a result, the students who participated in the activities of the first phase showed statistically significant improvements in their ability to have access to and verbally report the psychological lexicon appropriate to describe achievement emotions, compared to those who did not participate, supporting hypothesis 1a. Statistically significant effects demonstrated after the first phase of the intervention were shown to persist until the end of the research program.

Further, participating in the activities of the second phase was associated with increased ability to access and verbally report functional (particularly, reappraisal) strategies to regulate negative achievement emotions, supporting hypothesis 1b. Discussions with students suggested their appreciation of the relevance of being flexible in the use of ER strategies. It is worth noting that they recognized that in everyday life the same strategy can sometimes be functional and sometimes be dysfunctional depending on a variety of factors. This is in line with findings from the psychological literature which identifies a range of factors relating to situational characteristics or individual differences that can impact the efficacy of regulation strategies (Jacobs and Gross 2014). Unluckily, we were not able to assess long-term effects for improvements in ER knowledge, because of organizational issues due to school constraints. Future designs could be devised in order to assess long-term effects for all measured constructs.

We focused on strategies useful to regulate negative achievement emotions because of their high salience for students, as confirmed by the emotions that our participants indicated as disturbing them in activity 9. Previous research on the effects of mood and emotions on different psychological domains (Gobbo and Raccanello 2007; Fiedler and Beier 2014) has shown that positive emotions can also have disrupting effects. Compared to negative mood, positive mood is for example associated with lower accuracy and increased heuristic mistakes when the stimuli in a task must be processed in disciplined and careful ways. Future studies could be extended to foster ways to regulate them.

The efficacy of the intervention was further indicated by examining the possible generalization of the effects of the intervention to native language achievement. Correlations between native language achievement emotions and performance indicated that our data were in line with previous literature both on the structure of emotions and on the relation between emotions and performance (Pekrun et al. 2017). These descriptive data enabled us to validate the relations between the two constructs, i.e., achievement emotions and performance, as assumed by the CVT, to the Italian context, with a sample of middle school students. The analyses indicated that participating in the intervention was associated with lower intensity of negative emotions such as anxiety, anger, boredom, and hopelessness after the first phase, and anxiety and anger after the second phase, partially confirming hypothesis 2. Our experimental design did not allow us to examine further long-term effects, and the presence of final differences for only two emotions out of five does raise questions about the stability of the effects on emotions. It may be that external factors such as the end of the school year (at the end of the first phase) had an effect different compared to the end of the second phase, after which the students were facing their first state exam.

Regarding performance, we found improvements in native language grades only at the end of the intervention, and only for males, partially confirming hypothesis 3. On the one hand, the absence of differences between groups at the end of the seventh grade could be interpreted in light of the typical increasing trend in evaluations from the first to the second term of each year, as indicated by our data and anecdotally reported by some teachers, which could have acted as a confounding variable. On the other hand, while an improvement was observed in grades for males, females overall had better grades, and we might be observing some form of ceiling effect. In other words, it could have been difficult to detect the same increase that we identified for males amongst the females, given that there was not so much space for improvement in their grades. In addition, gender stereotypes, that is, expectations about females performing better in languages than males, and vice versa for natural sciences and math, may have played a role (e.g., Rowley et al. 2007). We could also speculate that the intervention might have triggered a sense of inter-gender competition amongst the males resulting in better grades. In any case, further data with larger samples and embedded in other domains should be gathered to generalize these effects due to interventions.emotional experience two years after an earthquake: An exploration

On the whole, findings on native language-related achievement emotions and performance are consistent with assumptions of the CVT (Pekrun 2006; Pekrun and Perry 2014), and for the first time, they corroborate what is described within Goetz and colleagues’ PEILAS model (Goetz and Bieg 2016; Goetz et al. 2005). Therefore, having interpreted such a model as a heuristic to foster the third component of EI in middle school students has provided an opportunity to gather empirical evidence on the relations between achievement emotions and performance in a domain-specific setting. Findings from this study could be considered as a preliminary step on the path to generalizing and operationalizing the assumptions of the PEILAS model in a variety of learning contexts.

In line with findings from a meta-analysis (Durlak et al. 2011), the overall efficacy of the intervention could be also linked to aspects of the format used, in which a school teacher participated in delivering most of the activities, and on the reliance on the so-called SAFE training procedures, and particularly the introduction of active learning techniques such as cooperative learning. Such techniques have the potential to foster a positive emotional climate and to affect self-regulation through the distribution of motivational and cognitive resources across the members of a group (Boekaerts and Corno 2005). Students’ engagement in the program activities, as anecdotally reported by the teacher, could be an indicator of the appeal of their content which was developed ad hoc for this intervention in order to fill in gaps in the paucity of age and domain-specific materials used in similar interventions (Goetz and Bieg 2016; Goetz et al. 2005).

While a positive climate could have been initially fostered by effects of novelty or cooperative learning settings, its maintenance could have been due to the way in which the order of activities had been planned, with potentially more stimulating and interesting activities incorporating social network communication or simulations presented at the end of each phase. On the whole, this positive climate could have in turn partially contributed to positive improvements in students’ achievement emotions and performance. At the same time, the teacher’s increased awareness on the role of emotions within the class could have had consequences at multiple levels, and it could have played a role in determining the documented changes. Future research should examine how environmental factors such as teachers’ emotional resources and/or awareness could change in parallel with emotional interventions directed at their students and influence students’ emotions along with other distal environmental antecedents postulated by the CVT.

On the whole, our findings supporting the utility of this intervention have several implications concerning effective ways to increase students’ emotional competence. One implication concerns the possibility of conducting this intervention again in other schools; modifying its contents to adapt to the specific characteristics of different schools, different classes, and different students. Our data confirming the links between increased abilities on understanding emotions and ER on the one hand, and achievement emotions and performance on the other hand, support the argument for schools to devote time and attention to interventions focusing on emotions. A second implication is that fostering students’ emotional competence can have positive consequences not only within the school: They could in fact be generalized to cope with the challenges presented both by everyday tasks external to the school and by extraordinary events like is the case for natural disasters such as earthquakes (Raccanello et al. 2020b) or pandemics (Raccanello et al. 2020c).

There were various limitations to this study. First, the measurement of achievement emotions was based on retrospective self-reports, with associated shortcomings such as social desirability bias and memory distortions (Pekrun and Bühner 2014). Future studies could go beyond these limitations including for example observational or physiological measures of emotions, or experience sampling methods, which are nevertheless much less economic and more time-consuming. However, self-report instruments still represent the most direct way to have insight into people’s emotional world, and we tried to balance social desirability by fostering a setting in which students could feel free to disclose their emotions (Pekrun and Bühner 2014). Second, we carried out the analyses at the individual level rather than at the classroom level because of our small sample size, due mainly to the length of the intervention. Consequently, we could not consider possible effects related to the nested nature of the data, limiting the causal inferences that can be drawn from our findings. This is however typical of school-based interventions conducted in the context of severely limited resources; something that has been shown to not negatively affect the quality of the outcomes (Schonert-Reichl et al. 2015). Future studies should explore whether the efficacy of similar interventions could be demonstrated with larger samples and with younger students, given the paucity of research on achievement emotions amongst these age groups (for exceptions, see Lichtenfeld et al. 2012; Raccanello et al. 2019). Third, the positive effects of the intervention on native language-related achievement emotions and performance could have been also linked to uncontrolled factors. For example, they could have been related to the cooperative learning setting fostered during the activities, associated in previous research with increased enjoyment and decreased boredom (Martínez-Sierra and García González 2014). Further studies could be conducted to disambiguate this issue, with a design requiring at least three groups: an experimental group engaged in activities fostering emotion understanding abilities through cooperative learning; a control group engaged in activities on topics different from emotions through cooperative learning; and another control group not engaged in any activity. Finally, the fact that the Italian teacher for the experimental group was different from the two Italian teachers for the control group could have acted as a confounding variable. Future research should try to take into account this issue when designing and implementing a randomized control trial. In our case, organizational constraints within the school excluded the possibility to exert any influence on the assignment of the students to the experimental or the control groups.

Despite these limitations, our findings indicated that an intervention focused on understanding emotions can be effective and have generalized influence on students’ achievement emotions and grades. Factors such as the use of valid experimental designs, the availability of age and subject-specific materials, or the engagement of teachers should be considered when planning emotion-focused school-based interventions at any school level. Gaining a clearer understanding of ways to assist children’s emotional development is an essential precursor to enabling their educational wellbeing.

## Electronic supplementary material

ESM 1 (DOCX 61 kb)
