# Supplementary material for: An intervention promoting understanding of achievement emotions with middle school students
Source: Eur J Psychol Educ. 2020 Oct 2;36(3):759–80. doi: 10.1007/s10212-020-00498-x (PMC7530548; doi:10.1007/s10212-020-00498-x)
Supplement: Supplementary file 1 — (DOCX 61 kb) [file 10212_2020_498_MOESM1_ESM.docx]

**Supplementary Materials**

**European Journal of Educational Psychology**

**An Intervention Promoting Understanding of Achievement Emotions with Middle School Students**

**Table A**

*Description of Objectives, Activities, Procedure and Materials for the Ten Units of the Intervention*

| Unit | Objective: Promoting knowledge on | Activity | Procedure and materials |
| --- | --- | --- | --- |
| 1 | Recognition of achievement emotions | 1. Recognizing emotional lexicon in narrative texts | The students worked in pairs. Each pair was presented ten extracts from ten narrative texts, in a fixed random order. Each extract was focused on one of the ten target achievement emotions, i.e., enjoyment (Dickinson, 2006), hope (Rowling, 2001), pride (Mastrocola, 2004), relief (Fine, 2000), relaxation (Bach, 1977), anxiety (Morgenstern, 2000), anger (Alcott, 2011), shame (Gatti, 2007), boredom (Maldini, 1994), and hopelessness (Pennac, 2008). To foster students to grasp the distinction between achievement emotions and emotions not referred to learning, six extracts referred to achievement emotions, and four to emotions not related to achievement (i.e., for hope, relaxation, relief, and anger). Each pair of students was proposed a written task in which they had to identify the main emotion described in each extract (i.e., *What is the main emotion to which the author refers?*); to identify the emotional terms used to describe that emotion (i.e., *Which words/expressions describe it? Circle them*); to state whether the described emotion was an achievement emotion or not (i.e., *Is the identified emotion an achievement emotion?*); and to report the cause of that emotion (i.e., *What is the main cause of the identified emotion?*). |
| 2 | Production of achievement emotions | 2. Using emotional lexicon in narrative texts | The students worked in small groups. They had to produce two brief narrative texts (i.e., *Together with your peers, write a brief narrative text including the achievement emotion assigned by the teacher. Beyond that specific emotion, you can use also synonyms, and you have to describe an event causing that emotion*), focused on a positive and a negative achievement emotion (with related causes) assigned by the teacher (i.e., pride and shame; relief and anger; enjoyment and hopelessness; hope and boredom; relaxation and anxiety). |
| 3 | Recognition of achievement emotions | 3. Recognizing emotional lexicon in expressive texts | The students worked individually. They were presented ten extracts from ten expressive texts (diary or letter texts), in a fixed random order. Each extract was written by the native language teacher, according to the following criteria: Being focused on one among the ten target achievement emotions; not including emotional terms; being in half of the cases focused on activity-related emotions and in the other half on outcome-related emotions; being not longer than 500 characters. The students were proposed a written task in which they had to identify the three synonyms describing the achievement emotion expressed in the text within a list of 30 terms (i.e., *The following terms, reported in random order, refer to the ten achievement emotions we worked on during the previous units. For each expressive text, indicate the three terms related to the main described emotion. Each term can be used only once*), and the cause of such emotion (i.e., *Then describe briefly the cause of each emotion*). |
| 4 | Production of achievement emotions | 4. Using emotional lexicon in expressive texts | The students worked individually. They were prompted to work on the ability to use the emotional lexicon through reflections on their personal experience. They were proposed open-ended questions on personally experienced frequent achievement emotions (i.e., *Among these ten achievement emotions, which is the emotion that you experience frequently at school? When?*), not frequent achievement emotions (i.e., *Among these ten achievement emotions, which is the emotion that you never experienced at school? Why?*), desirable achievement emotions (i.e., *Among these ten achievement emotions, which is the emotion that you would like to experience at school? When?*), and not desirable achievement emotions (i.e., *Among these ten achievement emotions, which is the emotion that you would not like to experience at school? Why?*). |
| 5 | Recognition of achievement emotions | 5. Recognizing emotional lexicon in dialogues | The students worked in small groups. Each group was presented ten Peanuts’ comic strips, in a fixed random order. Each strip focused on one of the ten target achievement emotions (Schulz, 2000, 2003). In order to make the task not monotonous, the emotional terms were reported in some cases but not in all of them (i.e., they were not reported for pride, hope, relief, anger, anxiety, and boredom). The students had to label the main emotion expressed within each strip (i.e., *What is the main emotion described?*) and to identify its cause (i.e., *What is the main cause of that emotion?*). They were also asked to identify which elements typical of comic language were used to express the identified emotion (i.e., *What are the elements typical of comic language that are used to express that emotion, i.e., facial expression of characters, gestures, onomatopoeias, typing?*). |
| 6 | Production of achievement emotions | 6. Using emotional lexicon in dialogues | In small groups, the students had to perform a brief sketch representing two events focused on two achievement emotions randomly assigned, choosing the context, the characters, the dialogues, the gestures. Each sketch had to be long no more than three minutes, and all the components of the group had to play a role. When the sketch was performed in front of the class, the other students had to answer individually to questions asking to identify the represented emotions (i.e., *Which emotion was represented?*), whether the represented emotion was an achievement emotion or not (i.e., *Was the represented emotion an achievement emotion?*), whether the gestures were effective (i.e., *Was the gestures effective?*), and whether the event was clearly represented (i.e., *Was the event clearly represented?*). |
| 7 | Recognition of achievement emotions | 7. Recognizing emotional lexicon in social network language | In small groups and then with the whole class, the students had to develop a shared code to represent the ten target achievement emotions using WhatsApp emoticons. WhatsApp is a cross-platform mobile messaging app which allows to exchange messages using texts, including a variety of images, among which emoticons. The students had to choose two emoticons, among 30 given ones, for each achievement emotion. Emoticons are stylized reproduction of facial emotional expressions used mainly within Internet communication to add non-verbal information to written communication. |
| 8 | Production of achievement emotions | 8. Using emotional lexicon in social network language | The students worked individually. They had to write dialogues using a simulated version of WhatsApp, including at least two achievement emotions with corresponding labels and emoticons, using the code developed in the previous unit. The simulated version of WhatsApp was a paper-and-pencil one, printed on a duplex A4 sheet, and a copy of the code was given as printed in another A4 sheet. |
| 9 | Recognition of achievement emotions in need of regulation | 9. Identifying emotions in need of regulation in different school settings | The students worked individually. They were asked to write at least one emotion that could disturb them for each of the proposed school setting, i.e., during lessons, before/during/after tests, and during homework (i.e., *For each situation,* *write at least one emotion that you can feel at school that can disturb you*). They were also asked to specify the subject domains in which they had felt the selected emotions. At the end, they identified which emotion disturbed them the most, and if they desired they shared it aloud. |
|  | Recognition of functional emotion regulation strategies | 10. Recognizing functional emotion regulation strategies | The students worked in pairs. They were presented a list of 19 emotion regulation strategies adapted from the literature (Phillips & Power, 2007) and they were asked to distinguish them as being functional or dysfunctional (i.e., *For each emotion regulation strategy, specify whether it is functional or not*). Among functional ways also reappraisal strategies were included (e.g., *I review my thoughts or beliefs*). At the end, the students shared in class how they had classified the strategies using post-its and building all together a poster that was affixed to the class walls. |
|  | Production of functional strategies to regulate achievement emotions | 11. Reporting verbally functional emotion regulation strategies in different school settings | The students worked individually. They were asked to think of a student that can feel achievement emotions that can disturb him/her, and to list at least one strategy for each of the five presented emotions (i.e., anxiety before a test, anger and sadness after a bad grade resulting from a test, shame during a test, and boredom during a lesson) that can help him/her to diminish the intensity or the duration of that emotion (i.e., *For each situation,* *write at least one functional strategy that can help to diminish the intensity or the duration of the proposed emotion*). The five situations were chosen because referred to settings for which each emotion is particularly intense (e.g., Authors, 2013; Authors, 2018), taking into account the distinction between prospective and retrospective emotions (e.g., Pekrun, 2006; Pekrun & Perry, 2014). |
| 10 | Recognition of functional strategies to regulate achievement emotions | 12. Selecting and negotiating functional emotion regulation strategies in different school settings | The students worked in small groups. They were asked to discuss together the emotion regulation strategies identified individually in the previous activity, to eliminate possible dysfunctional strategies, and to report in a poster those strategies evaluated as functional by all the members of each group. The strategies reported in the posters were shared together, and the researcher stimulated further reflections on the criteria used to select functional strategies and to delete dysfunctional ones. |
|  | Production of functional strategies to regulate achievement emotions | 13. Familiarizing with an instrument supporting the access to functional strategies to regulate achievement emotions in everyday life | The students worked at the class group level. The students were given a laminated bookmark including the picture of a traffic light including three terms corresponding to the three lights of a traffic light: *stop* in correspondence to the red light, *think* in correspondence to the yellow light, and *start again* in correspondence to the green light (adaptation from Morganti, 2012). The researcher prompted the students to reflect together on how this instrument could be useful to favour the access to emotion regulation strategies in everyday life (i.e., *You can use the traffic light every time in which you feel a disturbing emotion, and it helps to remember that you can do three things*). |
|  | Production of functional strategies to regulate achievement emotions | 14. Using functional strategies to regulate achievement emotions in a simulated situation | The students worked in small groups. They had to perform a brief sketch representing one event focused on one disturbing achievement emotion randomly assigned (i.e., anxiety before a test, anger and sadness after a bad grade resulting from a test, shame during a test, and boredom during a lesson), choosing the context, the characters, the dialogues, the gestures. They had to represent a functional emotion regulation strategy helping to cope with that negative emotion. Each sketch had to be long no more than three minutes and it had to be based on a written draft; all the components of the group had to play a role, as mime actors or voice-over. When the sketch was performed in front of the class, the other students had to give feedbacks on the efficacy in the representation of each strategy. |

**References (for supplementary materials)**

Alcott, L. M. (2011). *Piccole donne*. [Little women]. Einaudi.

Authors. (2013).

Authors. (2018).

Bach, R. (1977). *Il gabbiano Jonathan Livingston*. [Jonathan Livingston seagull]. RCS Rizzoli Libri.

Dickinson, E. (2006). *Lettere, 1845-1886*. [Letters, 1845-1886]. Einaudi.

Fine, A. (2000). *Quella strega di Tulip*. [That witch of Tulip]. LaFeltrinelli.

Gatti, F. (2007). *Viki che voleva andare a scuola*. [Viki who wanted to go to school]. Fabbri Editori.

Mastrocola, P. (2004). *Una barca nel bosco*. [A boat in the wood]. Ugo Guanda Editore.

Maldini, G. (1994). *La mia seconda prima.* [My second sixth-grade]. Edizioni EL.

Morganti, A. (2012). *Intelligenza emotiva e integrazione scolastica.* [Emotional intelligence and school integration]. Carocci.

Morgenstern, S. (2000). *Prima media*. [Sixth-grade]. Einaudi Ragazzi.

Pekrun, R. (2006). The control-value theory of achievement emotions: Assumptions, corollaries, and implications for educational research and practice. *Educational Psychology Review, 18*(4)*,* 315–341. https://doi.org/10.1007/s10648-006-9029-9

Pekrun, R., & Perry, R. P. (2014). Control-value theory of achievement emotions. In R. Pekrun & L. Linnenbrink-Garcia (Eds.), *International handbook of emotions in education* (pp. 120–141). Taylor and Francis.

Pennac, D. (2008). *Diario di scuola*. [School diary]. Giangiacomo Feltrinelli Editore.

Phillips, K. F. V., & Power, M. J., (2007). A new self-report measure of emotion regulation in adolescents: The regulation of emotions questionnaire. *Clinical Psychology and Psychotherapy, 14*, 145–156. https://doi.org/10.1002/cpp.523

Rowling, J. K. (2001). *Harry Potter e il calice di fuoco*. [Harry Potter and the globet of fire]. Salani.

Schulz, C. M. (2000). *Peanuts.* Arnoldo Mondadori Editore.

Schulz, C. M. (2003). *Peanuts.* Panini.
